# Comparison of Miller and Airtraq laryngoscopes for orotracheal intubation by physicians wearing CBRN protective equipment during infant resuscitation: a randomized crossover simulation study

**DOI:** 10.1186/s13049-016-0228-1

**Published:** 2016-03-22

**Authors:** Pierre-Géraud Claret, Renaud Asencio, Damien Rogier, Claire Roger, Philippe Fournier, Tu-Anh Tran, Mustapha Sebbane, Xavier Bobbia, Jean Emmanuel de La Coussaye

**Affiliations:** Department of Anesthesia Resuscitation Pain Emergency Medicine, Nîmes University Hospital, 1 place du Professeur Robert Debré, Nîmes, 30029 France; EA 2415, Clinical Research University Institute, Montpellier University, 641 Avenue du Doyen Gaston Giraud, Montpellier, 34093 France; Department of Pediatrics, Nîmes University Hospital, 1 place du Professeur Robert Debré, Nîmes, 30029 France

**Keywords:** Cross-over studies, Infant, Cardiopulmonary resuscitation, Intubation, Manikins, Protective devices, Accidents

## Abstract

**Background:**

The purpose of this study was to evaluate the performance of orotracheal intubation with the Miller laryngoscope compared with the Airtraq laryngoscope by emergency and pediatric physicians wearing CBRN-PPE type III on infant manikins with conventional airway. We hypothesized that in this situation, the orotracheal intubation with the Airtraq laryngoscope would be faster and more effective than with the Miller laryngoscope.

**Methods:**

This was a prospective, randomized, crossover, single-center study who recruited emergency department physicians on a voluntary basis. Each physician performed a total of 20 intubation trials while in CBRN-PPE with the two intubation techniques, Miller and Airtraq. Intubations by each airway device were tested over ten consecutive runs. The order of use of one or the other devices was randomized with a ratio of 1:1. The primary endpoint was overall orotracheal intubation success.

**Results:**

Fifty-five emergency and pediatric physicians were assessed for eligibility. Forty-one physicians were included in this study and 820 orotracheal intubation attempts were performed. The orotracheal intubation success rate with the Airtraq laryngoscope was higher than with the Miller (99 % vs. 92 %; *p*-adjusted <.001). The orotracheal intubation and glottis visualization times decreased with the number of attempts (*p* <.001). The median orotracheal intubation time with the Airtraq laryngoscope was lower than with the Miller laryngoscope (15 s vs. 20 s; *p*-adjusted <.001). The median glottis visualization time with the Airtraq laryngoscope and with the Miller laryngoscope were not different (6.0 s vs. 7.5 s; *p*-adjusted =.237). Thirty-four (83 %) physicians preferred the Airtraq laryngoscope versus 6 (15 %) for the Miller (*p*-adjusted <.001).

**Discussion:**

For tracheal intubation by physicians wearing CBRN-PPE during infant resuscitation simulation, we showed that the orotracheal intubation success rate with the Airtraq laryngoscope was higher than with the Miller laryngoscope and that orotracheal intubation time with the Airtraq laryngoscope was lower than with the Miller laryngoscope.

**Conclusions:**

It seems useful to train the physicians in emergency departments in the use of pediatric Airtraq and for the management of CBRN risks.

## Background

The rapid management of respiratory failure after exposure to a CBRN agent (nuclear, radiological, biological, and chemical) is a priority [1] to minimize the mortality rate [2, 3]. However, wearing CBRN personal protective equipment (PPE) generates constraints that inhibit the operational capabilities of the user [4]. Several studies have shown that wearing a CBRN garment increases the time required for orotracheal intubation (OTI) [2, 5–7]. Although the objective of the physician is to reduce the time of exposure to a toxic agent, stabilization of vital distress should not delay decontamination [1].

Both adults and children can be exposed to CBRN agents. For instance, the recent Ebola epidemic shows us that 14 % of patients are below 15 years of age [8]. Moreover, pediatric and infant OTI are difficult skills to learn and require continual practice to maintain competence and minimize the failure rate, regardless of the physician’s clinical background [9–11]. The Miller laryngoscope is commonly used for pediatric intubation; however, this device is difficult to use even for skilled professionals and could become detrimental in infant emergency situations [12].

The purpose of this study was to evaluate the performance of the Miller laryngoscope compared with the Airtraq laryngoscope for OTI by emergency and pediatric physicians wearing CBRN-PPE type III on infant manikins with conventional airway. We hypothesized that in this situation, the OTI with the Airtraq laryngoscope would be faster and more effective than with the Miller laryngoscope.

## Methods

### Trial design

This was a prospective, randomized, crossover, single-center study conducted at the University Hospital of Nîmes and approved by the local institutional review board (IRB N15/04.01).

### Participants

**Fig. 1:**
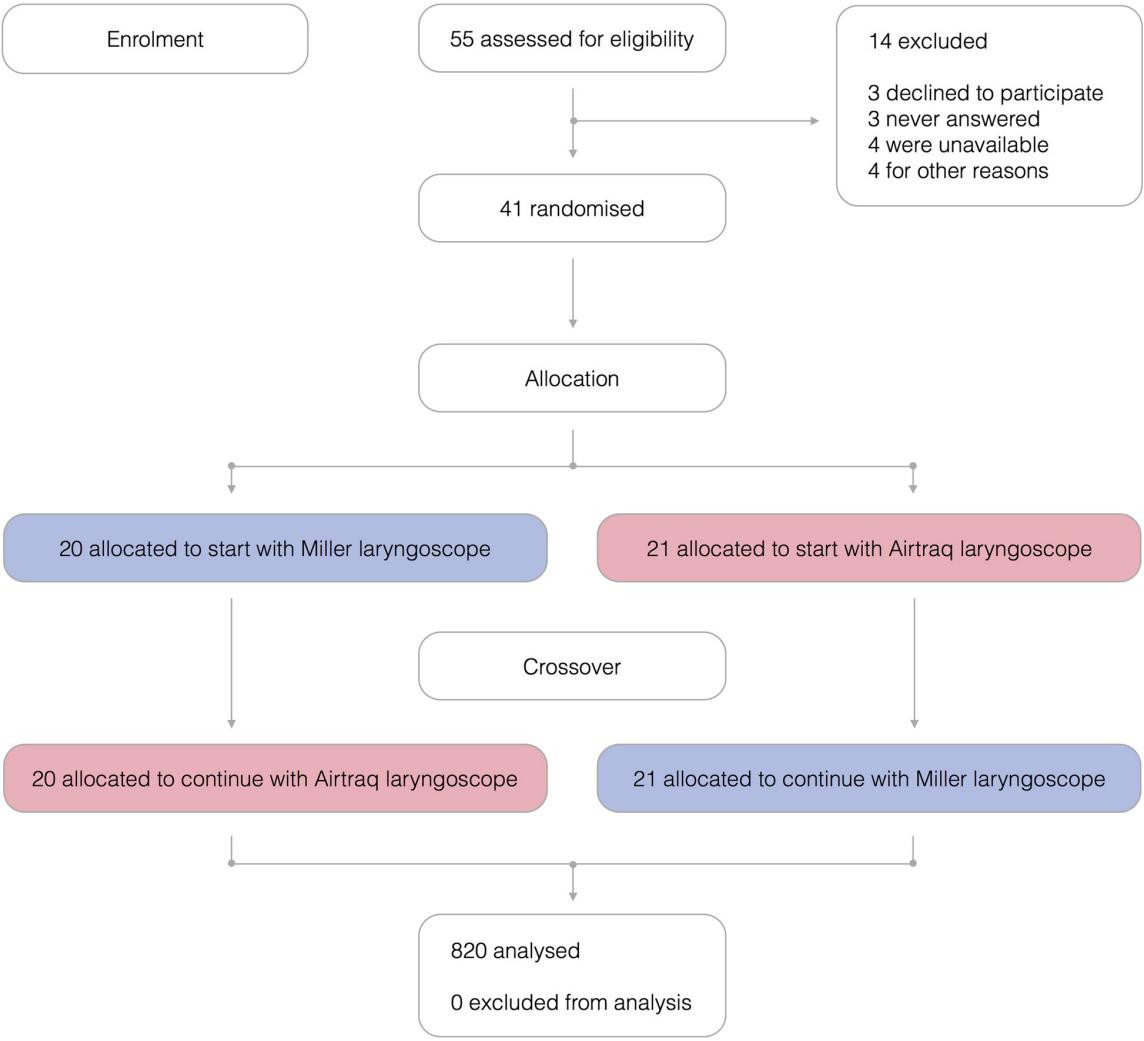
CONSORT flow diagram of design and recruitment of participants

Physicians included in the study were recruited on a voluntary basis. The inclusion criteria were that they had to be thesis doctors (Doctor of Medicine, MD or DM), fully trained and licensed (or in the course of validation) as an emergency medicine or pediatric physician. Short, standardized prior training was given to each physician in the wearing of the CBRN-PPE type III. We used for this study the AFNOR (Agence Française de Normalisation) type III CBRN-PPE, (Tychem F, Dupont, France), with hooded coverall, elasticated face, wrists, waist, and ankles. It is chemical protective type III (types 3-B, 4-B, 5-B, and 6-B) with butyl gloves. Masks were used with a gas and vapor cartridge filter type A2B2E2K2P3 (Fig. 1-A).

### Interventions

Each physician performed a total of 20 intubation trials while in CBRN-PPE with the two intubation techniques, Miller and Airtraq (Fig. 1). Intubations by each airway device were tested over ten consecutive runs. The order of use of one or the other devices was randomized with a ratio of 1:1 prior to the collection of data. Intubations were performed on a 3-month-old model Leardal Infant Airway Management Trainer (Laerdal Medical AS, Stavanger, Norway), with normal airway and with a 3.5 mm tube. Only one investigator collected data on a standardized form.

### Outcomes

The primary endpoint was overall OTI success. Overall OTI success was defined as successful OTI with the initial device, regardless of the number of attempts required. An attempt was defined as insertion of the laryngoscope into the manikin oropharynx irrespective of whether an attempt was made to pass the endotracheal tube. Successful OTI was defined as correct placement of the endotracheal tube in the trachea, as confirmed by the inflation of both lungs during blowing air and the visualization of the tube by the investigator. An intubation failure was defined by an intubation requiring a time greater than 120 s. In the case of esophageal intubation or a selective bronchial intubation, it was noticed to the physician who could then make another attempt; thus, it was not counted as an intubation failure. The secondary endpoints were the OTI time, glottis visualization time, and responses to a survey done at the end of each series. Orotracheal intubation time was defined as the time between when the physician has the laryngoscope in hand with the light on and when the endotracheal tube is connected to a bag valve mask with inflation of both lungs during blowing air. Glottis visualization time was defined as the time between when the physician has the laryngoscope in hand with the light on and when he/she visualizes the glottis. At the end of each of the two series of 10 tests intubation, each physician assessed the device used with a questionnaire of five items on ease of use (Q1), speed of handling (Q2), ease of insertion of the tube in oropharyngeal cavity (Q3) and through glottis (Q4), and visualization of the glottis and the vocal cords (Q5). The answer was given as verbal analog scale of 0–10 cm, with 0 representing “extremely easy or quick” and 10 being “extremely difficult or time-consuming.” A tenth question was personal preference between the two devices after use.

### Sample size and sequence generation

Based on our previous adult study [13], the following assumptions were made to calculate the number of patients to be included: we assumed an alpha risk of.05, a beta risk of.2. The overall OTI success with the classic laryngoscope was previously 78 %, the overall OTI success with the Airtraq was previously 98 %. We calculated that 40 participants would be required (paired, two-sided). Participants were randomized with a 1:1 ratio.

### Statistical methods

We described variables using percentages for qualitative variables and using median with interquartile range for quantitative variables. We compared qualitative variables by Fisher exact test and Kruskal-Wallis test. We compared quantitative variables by Student’s test. We studied the OTI and glottis visualization times evolution by analysis of variance. Multivariate logistic regression analyses were performed for each of the outcome variables: rate of successful OTI, OTI time, glottis visualization time, Cormack-Lehane score, number of attempts, number of required maneuvers, number of esophageal intubations, and number of selective bronchial intubations. The predictor variable of interest was intubation device. Other predictor variables were added to each model to adjust for confounding: physician specialty, emergency department experience, experience of infant intubation, and experience of wearing CBRN-PPE (*p*-adjusted). All statistical tests were two-sided. We considered as significant a *p*-value less than.05. We performed analysis using R version 3.1.2 (R Foundation for Statistical Computing, Vienna, Austria).

## Results

### Participant flow

**Fig. 2:**
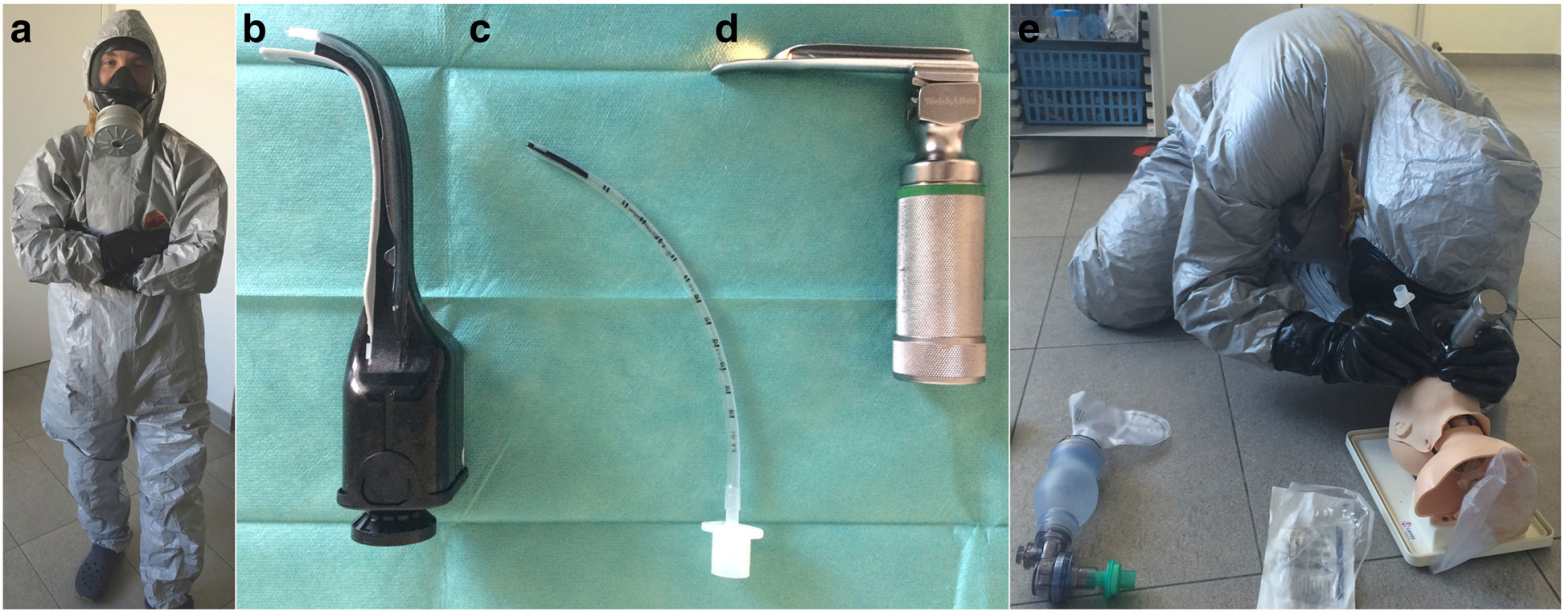
Physician wearing personal protective equipment (**a**), Airtraq laryngoscope (gray, size 0, mouth opening 11 mm) (**b**), infant endotracheal tube (outside diameter 3.5 mm) (**c**), Miller laryngoscope (size 1, length 105 mm) (**d**), and infant (3-month-old) cardiopulmonary resuscitation simulation (**e**)

Fifty-five emergency and pediatric physicians were assessed for eligibility. Forty-one physicians were included in this study and 820 OTI attempts were performed. The CONSORT diagram summarizing the flow of participants through the study is shown Fig. 2.

### Baseline data

The median [IQR] age of the physicians was 34 years [31–42]. Thirty-three (80 %) were emergency physicians and 8 (20 %) were pediatric physicians working at the pediatric emergency department. Among the emergency physicians, 25 (61 %) were holders of an emergency medicine diploma (DESC or CMU) and 7 (17 %) will validate their diploma in the current year. The overall median [IQR] ED working experience of the physicians was 5 years [2–13]. Forty-four (34 %) physicians had never intubated an infant and 16 (39 %) had never worn CBRN-PPE. The overall characteristics of the physicians are shown Table 1.

**Table 1:** Characteristics of participants

Characteristics	*N*= 41
Age, year (median, IQR)	34 [31–42]
Male, n (%)	20 (49)
Year of graduation (median, IQR)	2010 [2003–2014]
Diploma, n (%)	
EM specialty (DESC)	17 (41)
Last year of EM specialty	7 (17)
GP with EM specialty (CMU)	8 (20)
Pediatric specialty	8 (20)
Critical care specialty	1 (2)
Working experience in ED, year (median, IQR)	5 [2–13]
Experience of infant intubation, n (%)	
O	14 (34)
1 to 5	14 (34)
> 5	13 (32)
Experience of CBRN-PPE wearing, n (%)	
0	16 (39)
1 to 5	22 (53)
> 5	3 (8)
How comfortable are you (*) (median, IQR)	
When intubate infant	4 [2–6]
When wearing CBRN-PPE	4 [4–6]

*: verbal analogue scale from 0 to 10; CBRN-PPE: chemical, biological, radiological and nuclear personal protective equipment; CMU: capacité de médecine d’urgence (capacity of emergency medicine); DESC: diplômes d’études spécialisées complémentaires (complementary specialized studies diplomas); ED: emergency department; EM: emergency medicine; GP: general practitioner; IQR: interquartile range

### Outcomes

**Fig. 3:**
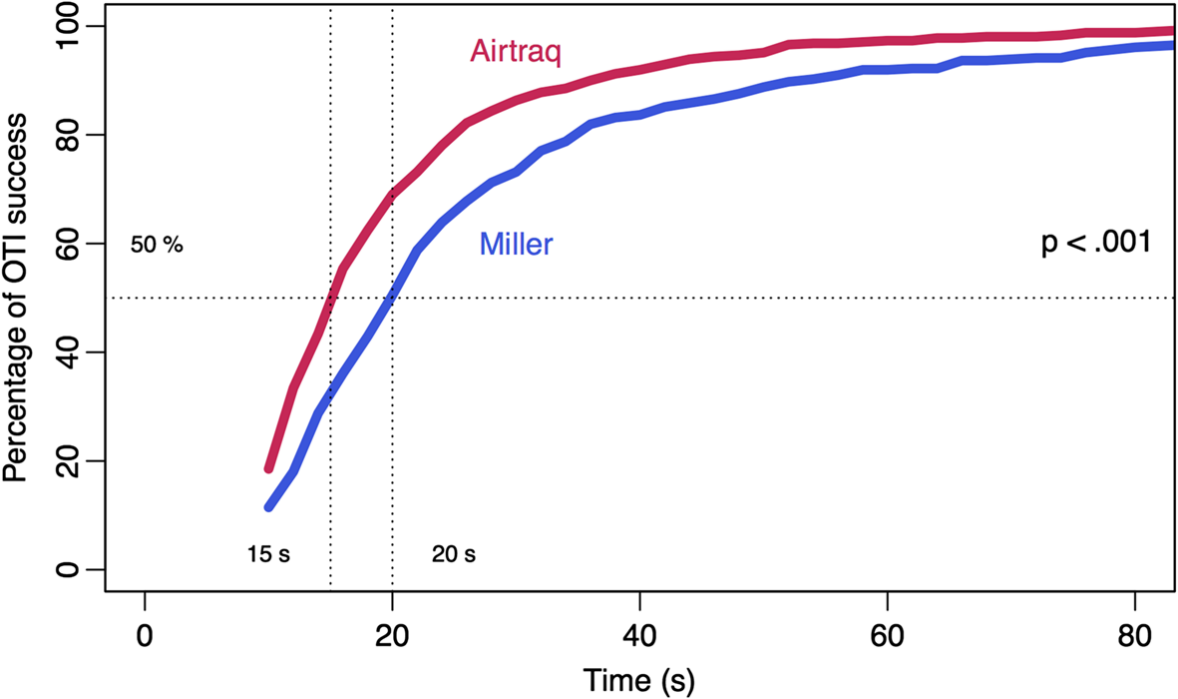
Percentage of orotracheal intubation (OTI) success in a given time using the Miller (*blue* curve) and Airtraq (*red* curve) laryngoscopes

The number of esophageal intubations and the number of optimization maneuvers were significantly lower with the Airtraq laryngoscope than with the Miller (99 % vs. 93 % (*p*-adjusted <.001) and 99 % vs. 95 % (*p*-adjusted <.001), respectively). There was more selective bronchial intubation with the Airtraq laryngoscope than with the Miller (94 % vs. 90 %; *p*-adjusted =.038). The OTI outcomes using the Miller and Airtraq laryngoscopes are summarized in Table 2 and Fig. 3 shows the percentage of orotracheal intubation success (i.e. correct placement of the endotracheal tube in the trachea, regardless of the number of attempts required) in a given time using the Miller (blue curve) and Airtraq (red curve) laryngoscopes.

**Table 2:** Orotracheal intubation outcomes using the Miller and Airtraq laryngoscopes

Characteristics	Miller (*N*= 410)	Aitraq (*N*= 410)	*p*-value (*)
Orotracheal intubation success, n (%)	376 (92)	408 (99)	<.001
Orotracheal intubation time, s (median, IQR)	20.0 [14.0–31.2]	15.0 [10.0–22.0]	<.001
Glottis visualization time, s (median, IQR)	7.5 [5.0–11.0]	6.0 [4.0–10.2]	.237
Cormack-Lehane score			<.001
1	196 (49)	362 (89)	
2	151 (38)	42 (10)	
3	26 (7)	4 (1)	
4	24 (6)		
Attempts, n (%)			.003
1	372 (92)	402 (98)	
2	25 (6)	7 (1)	
3	7 (2)	1 (1)	
Optimization manoeuvres required, n (%)			<.001
0	390 (95)	407 (99)	
1	17 (4)	3 (1)	
2	3 (1)	0 (0)	
Oesophageal intubations, n (%)			<.001
0	381 (93)	407 (99)	
1	24 (6)	3 (1)	
2	5 (1)	0 (0)	
Selective bronchial intubation, n (%)			.038
0	369 (90)	385 (94)	
1	41 (10)	25 (6)	

*: *p*-value adjusted for confounding included physician specialty, working experience in emergency department, experience of infant intubation, and experience of CBRN-PPE wearing; IQR: interquartile range

**Fig. 4:**
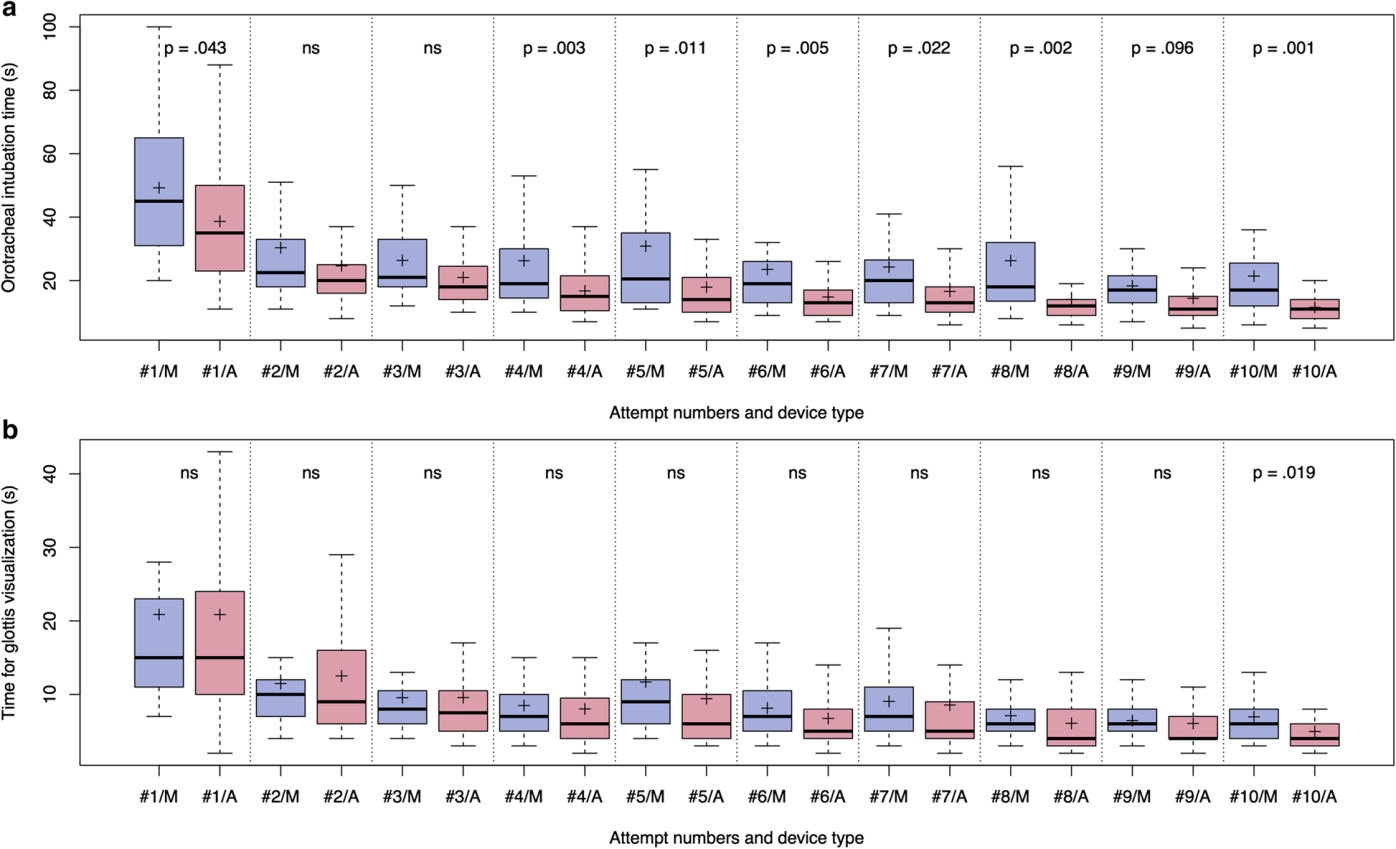
Orotracheal intubation (**a**) and glottis visualization times (**b**) using the Miller (*blue* boxplots) and Airtraq (*red* boxplots) laryngoscopes. Boxplots show mean (cross), median, interquartile range, and whiskers (defined as 1.5 times the value of the interquartile range). Orotracheal intubation time (**a**) was defined as the time between when the physician has the laryngoscope in hand with the light on and when the endotracheal tube is connected to a bag valve mask with inflation of both lungs during blowing air. Glottis visualization time (**b**) was defined as the time between when the physician has the laryngoscope in hand with the light on and when he/she visualizes the glottis. ns: non-significant

The OTI success rate with the Airtraq laryngoscope was higher than with the Miller (99 % vs. 92 %; *p*-adjusted <.001). The OTI and glottis visualization times decreased with the number of attempts (*p* <.001) (Fig. 4). The median OTI time with the Airtraq laryngoscope was lower than with the Miller laryngoscope (15 s vs. 20 s; *p*-adjusted <.001) (Fig. 4a). The median glottis visualization time with the Airtraq laryngoscope and with the Miller laryngoscope were not different (6.0 s vs. 7.5 s; *p*-adjusted =.237) (Fig. 4b).

**Fig. 5:**
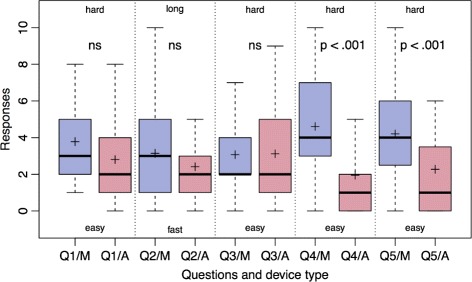
Survey responses, on a verbal analogue scale from 0 to 10, using the Miller (*blue* boxplots) and Airtraq (*red* boxplots) laryngoscopes. Questions were about ease of use (*Q1*), speed of handling (*Q2*), ease of insertion of the tube in oropharyngeal cavity (*Q3*) and through glottis (*Q4*), and visualization of the glottis and the vocal cords (*Q5*). Boxplots show mean (*cross*), median, interquartile range, and whiskers (defined as 1.5 times the value of the interquartile range) ns: non-significant

The Airtraq laryngoscope was the preferred device for the ease of tube insertion through the glottis (*p*-adjusted <.001) and for the glottis visualization (*p*-adjusted <.001). Thirty-four (83 %) physicians preferred the Airtraq laryngoscope versus 6 (15 %) for the Miller (*p*-adjusted <.001). One physician had no preference. The physicians’ survey responses are summarized in Fig. 5.

## Discussion

The objectives of our study were to evaluate the performance of OTI with the Miller laryngoscope compared with the Airtraq laryngoscope by emergency and pediatric physicians in protective CBRN-PPE type III, on infant manikins. Our study shows that the OTI success rate with the Airtraq laryngoscope was higher than with the Miller laryngoscope and that OTI time with the Airtraq laryngoscope was lower than with the Miller laryngoscope (independently from physician specialty, working experience in emergency department, experience of infant intubation, and CBRN-PPE experience).

In our study, OTI with the Airtraq laryngoscope is more effective, faster and easier than with the Miller laryngoscope. Indeed, the Airtraq device allows an intubation that is 5 s faster than the Miller device (*p*-adjusted <.001). Although this time difference is statistically significant, the clinical impact is probably low. Contrariwise, the success rate of intubation, which is also in favor of Airtraq, is more clinically significant. We assume that the display qualities of the Airtraq help explain these good results, especially in difficult intubation, such as in pediatric patients while wearing CBRN-PPE. Indeed, the anatomy of the infant makes the intubation process different than intubation in an adult patient. An infant has a smaller mouth, a shorter neck, a larger tongue, a less developed lower jaw and the larynx is situated higher. In CBRN risk situations, physicians must also wear protective clothing, which causes stress, loss of dexterity due to the use of butyl gloves [4, 6, 7] and a decrease in the visual field resulting from the use of the mask and the filter cartridge [2, 14]. Thus, the concept of difficult intubation includes not only difficulties due to the patient’s anatomy [15], but also difficulties caused by the clinical situation and the place of intubation [16]. Therefore, the anatomy of the infant, the lack of experience of physicians, and wearing of CBRN-PPE all contribute to make this intubation very difficult. Due to these characteristics, the Airtraq laryngoscope does not require alignment of the oral axis, the pharyngeal axis and the laryngeal axis. The use of the Airtraq does not require a movement of the tongue or a strong elevation of the epiglottis [17]. When the view of the glottis is centered and adjusted to the center of the screen, the endotracheal tube is guided by a channel through the vocal cords and pushed into the trachea. These benefits of the Airtraq seem even more important in very difficult situations, such as the one in our study. In our study, the Airtraq laryngoscope allows better visualization of the vocal cords (Fig. 5 Question 5, Cormack-Lehane score) leading to a better insertion of the endotracheal tube through the vocal cords (Fig. 5 Question 4). We assume this explains the better performance of the Airtraq and the lower selective intubation rate for esophageal and optimization maneuvers. In our study, 28 of 41 physicians (68 %) were not practiced in infant OTI (less than five OTIs during their career) and 16 of 41 (39 %) had never worn CBRN-PPE. We believe that this lack of experience explains the lack of comfort for pediatric OTI (4/10) and in wearing CBRN-PPE (4/10). However, despite this low comfort, it is reassuring that the OTI success rate was high. In addition, we observed a rapid stabilization of the Airtraq training curve.

Studies concerning intubation of infants in PPE are few. The following PubMed equation “(cbrn OR ppe OR hazmat OR ebola OR sars) AND (infant* OR child* OR pedia*) AND intubation” found only three outcomes, relatively distant from the subject of our study. However, we include below other studies in adults or in children (without the wearing of PPE) used to compare our results with those of the literature.

Concerning studies about adults and the success rate of OTI, the results are less reliable than our study since Woollard et al. [18], only used manikins with difficult airways and found a success rate significantly in favor of the Airtraq device. Further studies on manikins with normal airways have not found a significant success rate in favor of one or the other device [19, 20].

Concerning studies about adults and the OTI time, intubation with Airtraq is faster than the Macintosh as shown in studies by Di Marco [19], Woollard [18], Maharaj [20, 21], and Lu [22] in his meta-analysis (−14.79 s; CIs [ −25.13–4.46]). Another study of Castle highlighted that the loss of time during intubation while wearing CBRN-PPE was explained by more complicated movements and the loss of dexterity due to butyl gloves [23]. The absence of physician experience is confirmed by Woollard et al. [18] in normal outfit, and in the meta-analysis of Lu [22].

Concerning studies about adults wearing CBRN-PPE, our results partly confirm those of Castle et al. who compare six intubation techniques on adult manikins, first without and then with CBRN-PPE [23]. With CBRN-PPE, OTI time was 49.6 s ± 20.9 s for Macintosh versus 69.4 s ± 38.4 s for Airtraq and the intubation success rate was 92 % for Macintosh versus 91 % for the Airtraq device. However, when intubation is performed while wearing conventional care clothing, this conclusion is different, regardless of whether on manikins [18, 20, 21, 23], or on patients [19, 22] and regardless of the degree of difficulty of the airway or the level of experience of participants. Whereas some studies are partially consistent with ours, we previously found the opposite results in another study concerning adult manikins [13]. In that study, the Macintosh laryngoscope had a better success rate and a faster time to intubation than the Airtraq. We believe that these conflicting results reflect the difficulty in generalizing the adults’ results to children.

Concerning studies about children, the literature shows a superiority of the Airtraq as a conventional care outfit. Thus, Orliaguet et al. showed that the Airtraq had a shorter training curve and duration of intubation, with a lower failure rate [24]. Similarly, Riad et al. showed that in conventional care outfits, Airtraq was more efficient and faster than the Miller device, with a better rate of intubation and fewer optimization maneuvers [25]. On the other hand, White et al. showed no difference. None of these studies were conducted in CBRN-PPE.

In the present survey, we showed that the Airtraq is superior in terms of visualizing the glottis and the vocal cords. In a mannequin study involving post-graduate year emergency medicine residents, Aberle et al. showed that video laryngoscope with GlideScope had a higher perceived ease of use, but that traditional direct laryngoscopy was perceived to be more feasible for use in CBRN conditions [26]. Similarly, our study does not show better evaluation for the insertion of the probe in oropharyngeal cavity. Although we cannot exclude a type II error, this notion was also found with Dhonneur [27] since participants had noted that they could not properly direct intubation despite an excellent view of the vocal cords.

Our study was performed on a manikin; thus, generalization to clinical practice is difficult. Nevertheless, it seems useful to train physicians in emergency departments in the use of pediatric Airtraq. We also recommend adding pediatric Airtraq as equipment necessary for managing CBRN risks. Nevertheless, while Airtraq can be considered as a convenient tool for infant orotracheal intubation, this indirect vision device may not be useful in the presence of blood, vomit, or secretions in the oropharynx [28]. Further studies are needed to confirm our results. Our study and others highlight the specificity of pediatric care [24, 29]. These characteristics have implications for providing care in CBRN disasters, making resulting illness in children challenging to prevent, identify, and treat. The generalization of adult study results to pediatric care often leads to contradictory results. Future studies should be conducted specifically with children and infants. Ideally, these studies should be conducted in real situations. Furthermore, infant intubation is also performed by naso-tracheal route and future studies will compare naso-tracheal intubation to oro-tracheal intubation using the standard laryngoscope and the Airtraq model developed specifically for naso-tracheal intubation.

First, the simulations do not account for factors such as blood, vomit or secretions in the oropharynx. Second, the single-center nature of this study is also a limitation. Third, recruitment of participants was done on a voluntary basis, which causes a potential selection bias and a smaller sample of physicians. However, our crossover study partly eliminates this bias since we compared the performance of the same individual on both airway devices. Fourth, the collection of data does not allow us to adjust p-values to the fact that physicians had already intubated wearing PPE or with the Airtraq laryngoscope. Fifth, it may be applicable to move the infant because they are lighter than an adult. Finally, it is difficult to predict the applicability of the results of this study on patients because it is a study performed on a manikin. However, our methodology tries to adapt closer to real conditions. Nevertheless, it is important that further studies on patients are performed to confirm these results.

## Conclusions

For tracheal intubation by physicians wearing CBRN-PPE during infant resuscitation simulation, we found that the OTI success rate with the Airtraq laryngoscope was higher than with the Miller laryngoscope and that OTI time with the Airtraq laryngoscope was lower than with the Miller laryngoscope. It seems useful to train the physicians in emergency departments in the use of pediatric Airtraq and for the management of CBRN risks.

## Abbreviations

CBRN: nuclear, radiological, biological, and chemical
CMU: capacité de médecine d’urgence (capacity of emergency medicine)
DESC: diplô mes d’études spécialisées complémentaires (complementary specialized studies diplomas)
ED: emergency department
EM: emergency medicine
GP: general practitioner
IQR: interquartile range
ns: non-significant
OTI: orotracheal intubation
PPE: personal protective equipment
